# Knowledge, Awareness, and Perception of Common Eye Diseases and Eye Donation Among People Seeking Healthcare in a Tertiary Hospital in Telangana, South India

**DOI:** 10.7759/cureus.31412

**Published:** 2022-11-12

**Authors:** Mummareddi Dinesh Eshwar, Ayesha Jabeen, Quader Ahmed Jalily, Gulam Saidunnisa Begum

**Affiliations:** 1 Ophthalmology, Mahavir Institute Of Medical Sciences, Vikarabad, IND; 2 Biochemistry, Mahavir Institute of Medical Sciences, Vikarabad, IND; 3 Infectious Disease, Mahavir Institute of Medical Sciences, Vikarabad, IND; 4 Biochemistry, College of Medicine and Health Sciences, Sohar, OMN

**Keywords:** daly, national health programmes, avoidable blindness, knowledge & awareness, eye donation, health education & awareness, public health, glaucoma, cataract, corneal blindness

## Abstract

Background

Globally billions of people have vision impairment (VI) or blindness, and at least half of the VI could have been prevented or has yet to be addressed. With the policies focused exclusively on treating ailments, we need to recognize the need to educate the country's population regarding diseases and their outcomes. This is evident in the poor eye donation rates, as documented by the Eye Bank Association of India (EBAI). The National Programme for Prevention of Blindness (NPCB) also advocates the need for active campaigning to promote eye donations and improve the corneal procurement rate by increasing health awareness. This study aimed to assess the knowledge, awareness, and perception regarding eye diseases and eye donation among the rural population of Telangana, South India.

Methods

A cross-sectional study involving 150 participants who were randomly selected from non-triaged attendants in the outpatient queue at Mahavir institute of medical sciences (MIMS) was included. Trained enumerators used verbally administered, semi-structured questionnaires on their awareness and knowledge of cataracts, glaucoma, diabetic retinopathy, and night blindness. The Chi-square test was applied to determine the statistical significance of the results obtained from the pre-test and post-test. The significance threshold of the p-value was set at <0.05.

Results

The population studied belonged to a mean age of 34.98 years. The study included 72 (48%) male and 78 (52%) female subjects, and the majority (48.6%) of them belonged to the age range of 21-30 years. More than 85% of subjects belonged to the middle and lower middle class, and the majority (74.7%) were Hindus by religion. Regarding cataracts, 64 (42.7%) did not know, and 86 (57.3%) had varied perceptions. The awareness regarding glaucoma was the least (88.7%) of all common ocular diseases. The study showed a significant association between knowledge of eye diseases and literacy status plus the population's socioeconomic status (p<0.05 ). A significant association was found to exist between willingness to eye donation and the religion of the study population (p<0.05).

Conclusion

This study identifies that the awareness regarding various ocular diseases was poor. Moreover, the participants had an alarmingly high misconception regarding different aspects of eye donation. Increasing public awareness is essential to minimize eye diseases, improve eye care, and encourage eye donations.

## Introduction

Visual impairment (VI) and blindness are common public health problems worldwide. The World Health Organization (WHO) report enumerates the leading causes of VI: uncorrected refractive errors, cataracts, age-related macular degeneration, glaucoma, diabetic retinopathy, corneal opacity, and trachoma [1]. Interestingly, the Indian population suffers from VI majorly attributed to cataracts (62.6%), followed by refractive error (19.70%), glaucoma (5.80%), posterior segment disorder (4.70%), surgical complication (1.20%), and posterior capsular opacification (0.90%) [2].

Global estimates suggest that at least 2.2 billion people have some VI/blindness, of whom at least 1 billion have a VI that could have been prevented. The rates of VI due to various causes have shown a significant increase (285 million in 2017, 1.3 billion in 2018, and 2.2 billion in 2019) globally [1]. However, the scenario in India appears surprisingly different, with the prevalence rates showing a decreasing trend (1.1% in 2001/2002 to 1.0% in 2006/2007, and 0.45% in 2015-2018) as a result of a national strategic scheme, the National Program for Control of Blindness and Visual Impairment (NPCBVI) [2].

In the United Kingdom (UK), the burden due to VI and blindness has been increasing despite a fall in the total disability-adjusted life years (DALY) due to all causes. Since most vision-related problems are preventable, increasing investment in prevention and implementing early intervention strategies could improve the outcomes [3].

The WHO and the International Agency for the Prevention of Blindness (IAPB) jointly launched VISION 2020, which was aimed at eliminating avoidable blindness by the year 2020 [4]. Therefore, it is essential to understand the knowledge of populations regarding eye health and eye diseases. Awareness among people is depicted by a perception of facts and knowledge as a basic understanding of the etiology and symptoms of the disease [2]. Studies estimating the knowledge of eye care and diseases have been reported from different countries and the results of which form the foundation for eye health promotion [5-7]. Eye diseases resulting in VI/blindness are more common in elderly populations, and while getting care, people are acquainted with detailed knowledge about eye diseases [5]. Results from an Indian study show that the awareness and knowledge of common ocular conditions, such as cataracts, glaucoma, corneal blindness, trachoma, and diabetic retinopathy, were very poor [8].

Corneal blindness is one of the major causes [4th cause of blindness globally (5.1%)] for VI [1]. The cornea is a clear watch glass-like structure covering the eye's exterior. Corneal VI can be preceded by keratitis, corneal ulcer, trauma, bullous keratopathy, cataract surgery, keratoconus, corneal dystrophies, and trachoma. Corneal diseases can be attributed to infectious and inflammatory conditions that result in cornea scarring and ultimately lead to functional vision loss (opacification). Corneal blindness due to corneal opacification is treated/managed by corneal transplantation or keratoplasty. Corneal transplantation/corneal grafting is a surgical procedure wherein a donor corneal tissue/graft replaces the damaged or diseased cornea.

Awareness is far less among people residing in rural areas compared to urban residents. This may be due to various reasons like illiteracy, ignorance, poor access to health care, and various programs implemented by the government among the rural population. More than 83% of the Indian population is inhabited in rural areas, as per the 2011 census report, where there is chronic deprivation of basic health services exacerbated by the added challenge of VI [2]. Approximately 46% of all VI is avoidable/treatable, and without intervention in the health education rates, the number of individuals with VI might increase exponentially in the coming years. 

Therefore, this study was carried out to assess the knowledge and perception of eye diseases and eye donations among the rural populations residing in Telangana, South India.

## Materials and methods

A cross-sectional study was carried out for three months, August and October 2019. A simple random sampling method was used to recruit 150 study participants. The institutional ethics committee approved the study of the Mahavir institute of medical sciences (MIMS) (MIMS/IEC/2019/06), wherein all the participants duly explained the procedure, and informed consent was taken. The study participants were grouped based on the urban and semi-urban living environments.

All the subjects aged over 15 years and who gave informed consent were included in the study. Subjects under 15 years who had eye ailments and were under treatment were excluded from the study.

Data collection procedure

Well-trained enumerators were used to verbally administer a semi-structured and validated questionnaire that included their awareness, knowledge, and perception of cataracts, glaucoma, diabetic retinopathy, and night blindness. The questionnaire is structured into three sections. The first part included demographic details, the second contained awareness, perception, and knowledge of ocular diseases, and the third part included the awareness, knowledge, and perception of study participants on eye donation. Before the clinical evaluation, the investigator recorded the subjects' responses to the questionnaire. The eye diseases for which awareness was checked included cataracts, glaucoma, night blindness, and diabetic retinopathy. All the questions included were open-ended.

Statistical Analysis

The data obtained were entered into Microsoft Office 2019 Excel sheet (Microsoft® Corp., Redmond, WA), and statistical inferences were drawn using SPSS software version 24 (IBM Corp., Armonk, NY). The quantitative data were represented as percentages. The Chi-square test was applied to determine the statistical significance of the results obtained from the pre-test and post-test. The significance threshold of the p-value was set at <0.05.

## Results

A total of 150 subjects were randomly selected to participate in the study. The questionnaires on the awareness, knowledge, and perception regarding eye diseases and donations were administered in the local languages, and the investigators recorded the corresponding responses. The study included 72 (48%) male and 78 (52%) female subjects, and the majority (48.6%) of them belonged to the age range of 21-30 years. More than 85% of subjects belonged to the middle and lower middle class, and the majority (74.7%) were Hindus by religion. The study participants' demographic characteristic features, including socioeconomic status, educational qualifications, religion, and living environment, are delineated in Table 1.

**Table 1:** The demographic characteristics of study participants

Parameter	Variable	n (%) Total=150
Age (Years)	≤20	05 (3.3)
	21-30	73 (48.6)
	31-40	23 (15.4)
	41-50	25 (16.7)
	51-60	24 (16)
Sex	Male	72 (48)
	Female	78 (52)
Socioeconomic status	Upper	0 (0)
	Upper middle	5 (3.3)
	Middle	73 (48.7)
	Lower middle	60 (40)
	Lower	12 (8)
Religion	Hindu	112 (74.7)
	Muslim	29 (19.3)
	Christian	09 (06)
Educational status	Graduate	42 (28)
	High school	09 (06)
	Literate (informal)	40 (26.7)
	Illiterate	59 (39.3)
Living environment	Rural	82 (54.7)
	Urban	68 (45.3)

Out of 150 participants asked about cataracts, 64 (42.7%) did not know, and 86 (57.3%) had varied perceptions. Knowledge of cataracts was low among the study participants. However, few of them believed that cataract is an age-related eye disease and could present as a white spot/membrane on the eye. The knowledge of cataracts among the study subjects is shown in Table 2.

**Table 2:** Knowledge of cataracts among the study participants

Eye disease/cataract	Variable	n (%) Total=150
What is cataract	A white spot in the eye	20 (13.3)
	A lens change where the lens becomes opaque	08 (5.3)
	An age-related process leading to a decrease in vision	21 (14)
	A white membrane growing over the eye	37 (24.7)
	Don’t know	64 (42.7)
Source of information	Doctor/ophthalmologist/optometrist/optician	22 (25.6)
	Family member/friend/ relative suffering from it	54 (62.8)
	Others	10 (11.6)
How is a cataract treated?	Medicine	26 (33.3)
	Surgery	49 (56.9)
	Don’t know	11 (12.8)
Is cataract blindness reversible?	Yes	08 (9.3)
	No	58 (67.4)
	Don’t know	20 (23.3)
Do you know about Intraocular lens implantation?	Yes	13 (15.1)
	No	73 (84.9)

The awareness regarding glaucoma was the least of all common ocular diseases. Among those who were aware of glaucoma and none of them knew that vision loss due to glaucoma is reversible. The knowledge of glaucoma among the study participants is shown in Table 3.

**Table 3:** Knowledge of glaucoma among study participants

Eye disease/glaucoma	Variable	n (%)
What is glaucoma?	High pressure in the eye	08 (5.3)
	A disease where the nerve of the eye becomes weak	03 (02)
	An age-related process leading to a decrease in peripheral vision	06 (04)
	Don’t know	133 (88.7)
Source of information	Doctor/ophthalmologist/optometrist/optician	11 (64.8)
	Television, magazines, or other media	03 (17.6)
	Others	03 (17.6)
Disease prognosis	Permanent	04 (23.5)
	Reversible	0 (0)
	Don’t know	13 (76.5)

Compared to the knowledge of glaucoma, the awareness of diabetes-related vision problems was quite good among the study subjects. However, the knowledge concerning how eye diseases can occur and preventive measures could have been higher. The majority (53.3%) of participants did not know how regularly a diabetic person should get their eyes checked. The details of the study participants' knowledge of diabetes-related eye diseases are presented in Table 4.

**Table 4:** Knowledge of study participants about diabetes-related eye diseases

Diabetes-related eye disease	Variable	n (%)
Source of information	Doctor/ophthalmologist/optometrist/optician	13 (8.7)
	Eye camp	13 (8.7)
	Family member/friend/ relative suffering from it	08 (5.3)
Is diabetes-related visual impairment treatable?	Yes	34 (22.7)
	No	19 (12.7)
	Don’t know	97 (64.6)
Frequency of eye checkups required among diabetic patients	Once every six months	04 (2.7)
	Once a year	04 (2.7)
	Once in two years	07 (4.7)
	Don’t know	80 (53.3)

The awareness of night blindness was the highest among the study participants. However, many of them had several myths regarding night blindness. Moreover, the knowledge concerning the etiology and prevention of night blindness was considerably low, as depicted in Table 5.

**Table 5:** Awareness of study participants about night blindness

Eye disease/night blindness	Variable	n (%)
Common causes for night blindness	Vitamin deficiency	40 (26.7)
	Malnutrition	08 (8.7)
	Diarrhea	02 (5.3)
	Others	51 (34)
	Don’t know	44 (29.3)
Can night blindness be prevented in childhood?	Yes	50 (33.3)
	No	30 (20)
	Don’t know	70 (46.7)

Among the study participants, 58.7% knew/heard about eye donation. However, only 46.7% were willing to pledge their eyes to donations. Eye donation was considered a noble cause by 40.7% of study participants, and objections from family members were the most common cause that influenced their decision to donate their eyes. Most (62.7%) of the study participants believed that a full eye is used during transplantation. The details concerning the perception and knowledge of eye donation among the study participants are shown in Table 6.

**Table 6:** Awareness and knowledge of eye donation among study participants

Eye donation	Variable	n (%) Total=150
Have you heard about eye donation?	Yes	88 (58.7)
	No	62 (41.3)
Are you willing to donate your eyes?	Yes	70 (46.7)
	No	80 (53.3)
Factors promoting eye donation	Noble cause	61 (40.7)
	Please help the blind	33 (22)
	Inspired by an article/magazine	23 (15.3)
	Influenced by academic knowledge	30 ( 20)
	A friend received a corneal transplant	05 (3.3)
Reasons for not willing to donate eyes	Objection from family	53 (35.3)
	Body illtreated by organ transplant	33 (22)
	Health-related problem	04 (2.7)
	Age factor	12 (8)
	Dislike the basic idea of eye donation	23 (15.3)
	Religious restriction	33 (22)
	Not sure about the correct use of cornea after extraction	41 (27.3)
The ideal time for eye donation	Within two hours after death	4 (2.6)
	Within one day after death	34 (22.7)
	Within six hours after death	06 (4)
	Any time after the death	87 (58)
	Don’t know	19 (12.7)
Donated eyes are used for	Full transplant	94 (62.7)
	Corneal transplant	09 (6)
	Don’t know	47 (31.3)

The study participants' literacy rate and socioeconomic status significantly influenced their knowledge of cataracts, as shown in table 7. Similarly, the literacy rate and socioeconomic status significantly influenced the study participants' knowledge of glaucoma, as shown in Table 8. The knowledge of eye donation was significantly influenced by the literacy rate and socioeconomic status, as shown in Table 9. Interestingly, the study participants were noted to be significantly influenced by their religion, literacy, and socioeconomic status concerning the willingness to donate their eyes, as shown in Table 10. The knowledge about the fate of the donated eyes was marginally influenced by the literacy of the study participants as depicted in Table 11.

**Table 7:** The effect of religion, literacy, and socioeconomic status on the knowledge of cataracts *Statistically significant

Demographic factors	Variable	Knowledge about cataract (Total=150)		p-value
		Yes (86)	No (64)	
Religion	Hindu	62	50	0.4
	others	24	14	
Literacy status	Literates	65	26	0.0001*
	Illiterates	21	38	
Socioeconomic status	Lower and lower middle class	27	45	0.0002*
	Middle and upper class	59	19	

**Table 8:** Effect of religion, literacy, and socioeconomic status on the knowledge of glaucoma *Statistically significant

Demographic factors	Variable	Knowledge about glaucoma (Total=150)		p-value
		Yes (17)	No (133)	
Religion	Hindu	13	99	0.8
	others	04	34	
Literacy status	Literates	17	74	0.0004*
	Illiterates	0	59	
Socio economic status	Lower and lower middle class	0	72	0.0002*
	Middle and upper class	17	61	

**Table 9:** The effect of religion, literacy, and socioeconomic status on the knowledge of eye donation *Statistically significant

Demographic factors	Variable	Knowledge about eye donation (Total=150)		p-value
		Yes (88)	No (62)	
Religion	Hindu	62	50	0.1
	others	26	12	
Literacy status	Literates	63	28	0.0001*
	Illiterates	25	34	
Socio economic status	Lower and lower middle class	33	39	0.0002*
	Middle and upper class	55	23	

**Table 10:** The effect of religion, literacy, and socioeconomic status on the participants' willingness to donate eyes *Statistically significant

Demographic factors	Variable	Willingness for eye donation (Total=150)		p-value
		Yes (70)	No (80)	
Religion	Hindu	66	46	0.0001*
	others	04	34	
Literacy status	Literates	15	44	0.0002*
	Illiterates	55	36	
Socio economic status	Lower and lower middle class	23	49	0.0005*
	Middle and upper class	47	31	

**Table 11:** The effect of religion, literacy, and socioeconomic status on the knowledge regarding the fate of donated eyes *Statistically significant

Demographic factors	Variable	Knowledge about donated eyes (Total=150)		p-value
		Yes (103)	No (47)	
Religion	Hindu	73	39	0.1
	others	30	08	
Literacy status	Literates	68	23	0.04*
	Illiterates	35	24	
Socio economic status	Lower and lower middle class	44	28	0.06
	Middle and upper class	59	19	

## Discussion

More than 250 million people have blindness or a different form of VI globally [9]. According to a three-decade study (1980-2014) carried out by the Vision Loss Expert Group (VLEG) that included more than 90 countries, South Asia, including India, accounted for 18% of the burden, with more than 301 million people being affected by VI [10]. According to the data presented by the International Agency for the Prevention of Blindness (IAPB) Vision Atlas, people in the Eastern European region has the highest percentage (20.7%) of the crude prevalence of all kinds of VI [11]. Moreover, the number of aged people will increase because of the exponential increase in the world's population. This further will increase the burden of people suffering from VI or blindness.

The common eye diseases prevailing among people throughout the world include refractive errors like myopia (near-sightedness), hyperopia (farsightedness), and astigmatism (distorted vision at all distances), which usually affect children and young adults. Presbyopia is a visual defect that generally affects middle-aged people. Other eye diseases commonly seen are age-related macular degeneration, cataracts, glaucoma, diabetes-related retinopathy, amblyopia (lazy eye), and night blindness, among others [12].

Since most eye diseases are preventable, the World Health Organization (WHO) has proposed implementing the integrated people-centered eye care (IPEC) model. This includes devising tools to diagnose, treat, and manage common eye diseases and using mobile toolkits to increase awareness, bring health literacy about reducing the risk factors for preventable diseases, and increase compliance regarding regular eye examinations [1]. 

The awareness of cataracts, glaucoma and diabetic retinopathy among the study participants was majorly attributed to a family member, friend, or relative suffering from a respective eye disease. Awareness of cataracts was higher among the subjects aged above 50 years, and Muslims were least convenient to speak about eye donation because of religious reasons. Despite the satisfactory awareness of cataracts being good, knowledge of cataracts was inadequate. Of those aware of cataracts, 37% expressed cataracts as a white membrane. This is still reasonable because 64% of them were unaware and had no knowledge of cataracts. Most subjects were aware that cataract treatment is surgery but was unaware that sight can be restored after an intervention. Of those aware of cataracts, 84.9% have not heard of intraocular implantation. The awareness of cataracts reported in this study was similar to a most recent study from the same geographical region, and the awareness levels correlated with educational status [13].

Understanding of glaucoma among the study population was remarkably poor. A recent study from Orissa, India, noted that inaccessible healthcare facilities, social/cultural beliefs, and low socioeconomic conditions have contributed to negligence in seeking eye care among people [14]. Early detection of glaucoma can prevent the advancement of the disease. However, because of the lack of significant symptoms, early diagnosis is challenging, which can be compensated for by regular eye testing. 

Knowledge and awareness of night blindness were moderate in the study population. A majority (51%) of the participants revealed other causes like consanguinity and heredity as the common reasons for night blindness. Although night blindness in childhood is attributed to vitamin A deficiency, many (46.7%) participants from this study were unaware. Lacking knowledge of the causes of night blindness in children could predispose them to such diseases. Increasing awareness and adequate supplementation of vitamin A can prevent night blindness. 

Millions worldwide suffer from diabetes mellitus each year in India and elsewhere. The study participants' awareness of diabetes-related eye diseases was low, and better socioeconomic status correlated with improved knowledge of diabetic retinopathy. This may have been because moderate to high-income people have increased medical and diagnostic care access. Among those who were aware that diabetes causes VI, only 2.7% responded that an eye checkup should be performed every six months. In comparison, 53.3% responded to the frequency of eye checkups whenever necessary. The prevalence of diabetic retinopathy in India was around 16%, as evidenced by the results of a previous study [15].

Corneal transplantation offers the potential for sight restoration to those blind from corneal diseases. This, however, depends on people willing to pledge their eyes for donation and relatives willing to honor that pledge upon the person's death [16,17]. Data from our study suggest additional efforts to improve awareness of eye donation in the community. It is a matter of concern that only 58.7% of the subjects heard about eye donation, 6% knew about corneal transplantation, and only 4% understood when to donate their eyes. The timing of eye donation is essential since the eyes donated later than 6 hours after death cannot be used for transplantation. Also, a sizeable proportion of the population needs to be made aware of how donated eyes are used and possibly needs to understand the potential for sight restoration that corneal transplantation offers. Lack of this essential knowledge and facts could hinder eye donation for many willing to pledge their eyes.

A significant proportion of current awareness of eye donations has been through publicity campaigns run by non-government organizations (NGOs) and other voluntary groups and a minor contribution by media campaigns by government agencies. The National Health Promotion (NHP), India, observes National Eye Donation Fortnight every year between 25th August and eight September to promote eye donations [18]. Moreover, Coronavirus Disease-19 (COVID-19) significantly affected knowledge of eye care and donations, as evidenced by a 63% reduction during the pandemic [19]. 

Study limitations

This study was limited to a specific geographical region and included a small proportion of people, and it only assessed the level of awareness concerning a few eye diseases. The population studied included those residing in rural and semi-urban environments; data on the urban population was not obtained.

## Conclusions

Although some progress has been made in reducing blindness in India, further improvements necessitate increasing the community's health literacy. The results of the current study indicate a pressing need for health education in the study population to improve awareness and understanding of eye diseases and eye donation. Our findings suggest that health literacy and public health interventions for eye care should target specific groups of the population, particularly those with lower education and socioeconomic status. This is especially important in developing countries like India, where most of the population resides in rural areas. Growing awareness and knowledge of common eye disorders and eye donation could contribute to an improved acquaintance that favors routine eye examination among people. This enables early diagnosis and treatment, avoids VI/blindness, and minimizes the cost of eye care. Health education from early life could positively impact individuals who otherwise develop wrong perceptions and believe false information about preventable eye diseases. Regional camps and workshops are necessary to educate people about disorders that cause blindness in the long run and those which are irreversible and require preventive measures. The myths and superstitious beliefs about eye diseases and eye donation among people require serious intervention to achieve complete vision rehabilitation and restoration.

## Appendices

Questionnaire-1 (DEMOGRAPHIC DETAILS)

**Table 12:** Demographic characteristics

Parameter	Variable
Name	
Age	
Sex	Male
	Female
Occupation	
Socioeconomic status	Upper class (≥36000$ per annum)
	Upper middle class (15,000-35000$ per annum)
	Middle class (5000-14000$ per annum)
	Lower middle class (1000-5000$ per annum)
	Lower (<1000$ per annum)
Religion	Hindu
	Muslim
	Christian
	Others
Educational status	Graduate
	High school
	Literate (informal)
	Illiterate

Questionnaire-2 (OCULAR DISEASES AWARENESS)

Tick the appropriate responses to the questions below:

**Table 13:** Cataract Questionnaire

Cataract				
					What is a cataract?	How did you come to know about cataracts?	How is it treated?	Is it possible to get back vision from cataract blindness?	Do you know about intraocular lens implantation?
a) A white spot in the eye	a)Doctor/ ophthalmologist/ optometrist/ optician	a) By medicines	a) No	a) No
b) A lens change where a lens becomes opaque	b) Eye camp	b) By Surgery	b) Yes	b) Yes
c) An age-related process leading to a decrease in vision	c) Family member/friend/ relative suffering from it	c) Do not know	c) Don’t know	
d) A white membrane growing over the eye	d) Television/magazines /other media	d) Others		
	e) Others			
1. Are you aware of any central and state schemes for ocular health? Yes/No				
2. If the response is YES what all schemes have you been associated with?				
a)				
b)				
3. Did you attend any camps organized by medical colleges/hospitals/NGOs? Yes/No				
4. Are you aware of the state scheme “Kantivelugu” scheme? Yes/No				
5. If so have you been to the nearest center? Yes/No				

**Table 14:** Glaucoma Questionnaire

GLAUCOMA		
What is glaucoma?	How did you come to know about glaucoma?	Glaucoma is??
1. High pressure in the eye	1. Doctor/ ophthalmologist/ optometrist/ optician	1. Permanent
2. A disease where the nerve of the eye becomes weak	2. Family member/ friend/ relative suffering from it	2. Reversible
3. Damage to the nerve of the eye due to high pressure	3. Family member/friend/relative not suffering from it	3. Don't Know
4. An age-related process leading to a decrease in peripheral vision	4. Television, magazines, or other media	
	5. Others	

**Table 15:** Diabetes and loss of Vision questionnaire

DIABETES AND DECREASE IN VISION			
How did you come to know about it?	Is the decrease in vision due to diabetes treatable?	How frequently should a person with diabetes go for an eye checkup?	
1. Doctor/ ophthalmologist/ optometrist/ optician	1. No	1. Once every 6 months	
2. Family member/friend/relative suffering from it	2. Yes	2. Once a year	
3. Family member/friend/relative not suffering from it	3. Don’t know	3. Once every 2 years	
4. Television, magazines, or other media		4. Depending on how much vision has been affected by diabetes	
5. Others		5. Don’t know	

**Table 16:** Night Blindness Questionnaire

NIGHT BLINDNESS	
What is the common cause of night blindness during childhood?	Can night blindness during childhood be prevented?
1. Vitamin deficiency	1. No
2. Malnutrition	2. Yes
3. Diarrhoea	3. Don’t know
4. others	

QUESTIONNAIRE-3 (EYE DONATION PERCEPTION ASSESSMENT)

**Table 17:** Eye Donation Questionaire

Heard about eye donation?	Yes	No
Willingness for eye donation?	Yes	No
What are promoting factors for eye donation according to you		
FACTORS	RESPONSE	
Noble cause	Yes	No
A pleasure to help the blind	Yes	No
Monetary benefits	Yes	No
Inspired by an article/magazine	Yes	No
Influenced by knowledge in academics	Yes	No
Friend received cornea	Yes	No
Friend received cornea	Yes	No
Reasons for non-willingness for eye donation		
REASON	RESPONSE	
Objection from family	Yes	No
Body illtreated by organ transplant.	Yes	No
Health-related problem	Yes	No
Age factor	Yes	No
Dislikebasic idea of eye donation	Yes	No
Religious restriction	Yes	No
Not sure about the correct use of cornea after extraction	Yes	No
Knowledge about the ideal time for eye donation		
TIME AFTER DEATH	RESPONSE	
Within 2 hrs	Yes	No
Within 1 day	Yes	No
Within 6 hrs	Yes	No
Anytime after death	Yes	No
Knowledge about donated eyes		
DONATED EYES ARE USED FOR	RESPONSE	
Transplant full	Yes	No
Corneal transplant	Yes	No
Lens transplant	Yes	No
